# Clinical Significance of Isolated Anti-HBc Positivity in Cases of Chronic Liver Disease in New Delhi, India

**DOI:** 10.4103/0974-777X.52978

**Published:** 2009

**Authors:** Manisha Jain, Anita Chakravarti, P Kar

**Affiliations:** *Department of Microbiology and Maulana Azad Medical College, New Delhi, India*; 1 *Department of Medicine, Maulana Azad Medical College, New Delhi, India*

**Keywords:** Isolated anti-HBc IgG, HCV co-infection, Liver disease

## Abstract

**Background:**

The presence of anti-HBc IgG in the absence of HBsAg is usually indicative of a past self-limiting HBV infection. But it is frequently associated with co-infection with HCV which can worsen the existing status of chronic liver disease (CLD).

**Objectives:**

The present study was planned to evaluate the significance of isolated HBc IgG positivity in patients of CLD and look for the presence of HCV co-infection in such patients.

**Methods:**

Clinical profiles and biochemical tests were done for all the 77 CLD cases included in the study. Blood samples were taken from these patients and tested by the commercially available EIA for the presence of HBsAg, anti-HBc IgG, anti-HBs and anti-HCV. HBV DNA was detected by amplifying the surface region in all the cases.

**Results:**

Isolated anti-HBc IgG positivity defined as the presence of anti-HBc IgG in absence of any other serological markers of HBV infection was detected in 28 patients. Out of 64 patients positive for anti-HBc IgG 36 had the markers of HBV, either HBsAg, HBV DNA or anti-HBs alone or in combination. There was a significant association between isolated anti-HBc IgG positivity and HCV co-infection.

**Conclusion:**

Anti-HBc IgG should be tested in all patients with CLD as it is frequently the only marker of HBV infection in such patients and they should be monitored closely as such patients can develop CLD. Presence of co-infection with HCV should be actively searched for in such patients.

## INTRODUCTION

Both Hepatitis B virus (HBV) and Hepatitis C virus (HCV) are the major causes of chronic liver disease (CLD) worldwide.[1,2] The diagnosis of Hepatitis B infection by convention depends on the presence of HbsAg but in 10–20% of patients only anti-HBc IgG can be detected which is usually indicative of past self-limiting HBV infection.[2–4] The clinical significance of such a finding is being recognized and it is seen that even in HbsAg and anti HCV negative patients CLD can develop and such patients show anti-HBc IgG as the only marker.[2–4] Isolated anti-HBc IgG is also frequently observed in intravenous drug abusers, HIV infected individuals, HBV and HCV co-infected patients and pregnant females.[3,4]

In cases of co-infection with both HBV and HCV serious consequences are seen with more severe and aggressive liver disease.[2] Prevalence of hepatocellular carcinoma (HCC) is higher in patients of co-infection than a single infection.[5] Both HBV and HCV interact with each other in a poorly defined way and so the clinical and virological profile varies in cases of HBV and HCV co-infection. Studies have shown that either one of these viruses may predominate during the clinical course of co-infection.[6–8] The clinical significance of the silent HBV co-infection in HCV associated CLD has not been fully understood. Though the role of HBV in HCV associated HCC patients who are negative for hepatitis B surface antigens (HbsAg) remains poorly defined, high prevalence of HBV markers like HBc IgG is seen in patients with HCV related chronic liver disease, particularly those with hepatocellular carcinoma, suggesting that H BV infection, probably including latent infection, may play an important role in carcinogenesis in these patients.[9]

Epidemiologic results have also shown that prior exposure to HBV infection is a risk factor for the development of HCC.[10] Hence in HBV-endemic areas, the possibility of co-infection of HBV in HbsAg-negative patients with HCV infection should be considered. The present study was thus planned to evaluate the significance of isolated HBc IgG positivity and look for the presence of HCV co-infection in such patients.

## MATERIALS AND METHODS

The present study was conducted in the Departments of Microbiology and Medicine, Maulana Azad Medical College and associated Lok Nayak and GB Pant hospitals in New Delhi from January 2003 - March 2004.

The study group included a total of 77 cases of chronic liver disease selected randomly from the Medical Outpatient Department or Medical Wards of Lok Nayak Hospital. All the three types of CLD i.e. chronic hepatitis, cirrhosis and hepatocellular carcinoma were included and were diagnosed on the basis of clinical, biochemical, serological and/ or histopathological study. Informed consent was taken from these patients before their inclusion in the study. The patients were evaluated on the basis of a detailed history and detailed physical and systemic examination. Autoimmune hepatitis, Wilson's disease, primary hemachromatosis and other infective causes besides viral hepatitis could not be ruled out.

Seven ml of blood was collected aseptically from all patients and it was subjected to a battery of biochemical and hematological investigations. Serum for viral serology was stored at −20°C until further testing was done. Serological tests were performed using commercially available ELISA kits according to the manufacturer's instructions. The various serological tests performed in all the patients included HBsAg provided by BIORAD, antibody to the core antigen, (HBc IgG) using commercially available ELISA kits by Biochem Immunosystems and antibody to Hepatitis C Virus (anti HCV) by Dia Sorin. Anti HBS was tested in all the patients using ELISA kit by SRL.

Polymerase chain reaction was performed in all the 77 samples for HBV DNA. HBV DNA was extracted from the serum sample using the standard phenol-chloroform-isoamylalcohol method.[11] PCR was carried out for the amplification of a 321 base pair sequence of the surface gene of HBV. The amplified products were detected by running 8-10 μl of the products along with the molecular weight marker (f × 174 Hae *III* digest) on 3% agarose gel stained with ethidium bromide and the 321 base pair product was compared with the known DNA marker (f × 174 Hae *III* digest) under UV illumination. The results were calculated according to manufacturer's instruction. The results were statistically analyzed using Chi square test and Student's *t*-test. *P* value of < 0.05 was considered as statistically significant.

## RESULTS

A total of 77 cases of chronic liver disease were included in the study. The cases were divided as having either chronic hepatitis, cirrhosis or hepatocellular carcinoma. Since other etiology of chronic liver disease besides hepatitis markers was not specifically looked for, hepatitis B virus was the most common cause which could be assigned to 83.1% based on the presence of HBc IgG. Presence of various serological markers in each group is given in Table 1. Surface antigen to HBV could be detected in 28 cases i.e. 36.36% whereas 64 cases (83.11%) had the presence of HBc IgG. HBV DNA was detected in 14 patients, of which 13 were HBsAg positive and one was negative for HbsAg; this patient had HCV co- infection. Antibody to surface antigen (anti-HBs) was present in 23.37% (18/77) cases. Seven patients of the 35 cases (20%) having HBc IgG as the only marker of HBV infection also had the presence of anti-HBs. Thus isolated anti-HBc IgG positivity was seen in 28 cases. The prevalence of HCV infection on the basis of anti-HCV positivity was 15.58% (12/77 cases). Anti-HCV could not be detected even in a single case of HBsAg positive patients. All the three cases of HCC had evidence of HBV infection either past or present and two of these had presence of HCV co-infection.

**Table 1:** Demographic profile and various viral markers in patients of chronic hepatitis, cirrhosis and hepatocellular carcinoma

	Chronic hepatitis (n = 21) (%)	Cirrhosis (n = 53) (%)	HCC (n = 3) (%)	Total
Age (years)	46.81 ± 12.15	46.6 ± 14.48	49.3 ± 14.48	77
Sex (Male: Female)	13:8	40:13	3:0	77
HBsAg	7 (33.3)	20 (37.7)	1 (33.3)	28
Anti-HBc IgG	16 (76.1)	45 (84.9)	3 (100)	64
Anti-HCV	3 (14.2)	7 (13.2)	2 (66.7)	12
HBV DNA	2	11	1	14
Anti HBs	6	11	1	18

Statistical analysis done using Chi square test, HCC - Hepatocellular carcinoma

Out of 28 cases of isolated anti-HBc IgG positive patients as many as eight also had the presence of HCV co-infection (28.57%). On comparing the prevalence of HCV co-infection in HBsAg positive and isolated anti-HBc IgG positive patients, a statistically significant association was seen between isolated anti-HBc IgG seropositivity and HCV co-infection with *P* value being < 0.05. The patients showing isolated anti-HBc IgG were further grouped into those having HCV co-infection (8 cases) and those who did not have HCV co-infection (20 cases). Clinical parameters and the biochemical profile among both the groups was similar and there was no statistically significant difference among the groups [Tables 2 and 3].

**Table 2:** Demographic and clinical profile of patients with isolated anti-HBc IgG positivity in relation to hepatitis C virus

Clinical presentation	HCV positive (n = 8) (%)	HCV negative (n = 20) (%)	*P*-value*
Age	46.22 ± 11.7	48.5 ± 12.6	NS (>0.05)
Abdominal discomfort	8 (88.8)	12 (85.7)	NS (>0.05)
Fever	7 (77.7)	10 (71.4)	NS (>0.05)
Anorexia	6 (66.6)	10 (71.4)	NS (>0.05)
Prodromal symptoms	6 (66.6)	9 (64.2)	NS (>0.05)
Jaundice	6 (66.6)	11 (78.5)	NS (>0.05)
Nausea	6 (66.6)	8 (57.1)	NS (>0.05)
Vomiting	6 (66.6)	7 (50)	NS (>0.05)
Hematemesis	3 (33.3)	7 (50)	NS (>0.05)
Pruritus	3 (33.3)	5 (35.7)	NS (>0.05)
Malena	2 (22.2)	6 (42.8)	NS (>0.05)
Clay stools	0	1 (7.1)	NS (>0.05)

* Statistical analysis done using Chi square test

**Table 3:** Biochemical profile in patients with isolated anti-HBc positivity in relation to hepatitis C virus

Parameter	HCV positive (n = 8)		HCV negative (n = 20)		*P*-value NS (>0.05)
			
	Mean	SD	Mean	SD	
SGOT (U/mL)	46	24.55	104.14	202.87	NS (>0.05)
SGPT (U/mL)	53	53.22	98.43	204.23	NS (>0.05)
ALP (U/mL)	21.44	10.88	44.93	84.87	NS (>0.05)
Total bilirubin (mg %)	3.9	4.12	5.08	6.30	NS (>0.05)
Direct bilirubin (mg %)	2.08	2.36	3.16	4.56	S (0.034)
Total proteins (gm %)	5.93	1.11	6.07	1.09	NS (>0.05)
Albumin (gm %)	3.19	0.68	3.13	0.44	NS (>0.05)

* Statistical analysis done using Student's *t*-test

## DISCUSSION

Surface antigen of HBV (HBsAg) by convention is used as the only marker to diagnose the prevalence of HBV infection, but this can miss a substantial number of cases. Additional tests like anti-HBc IgG and HBV DNA can help identify further cases as is seen in the present study too.[10,11] The prevalence of HBV infection taken solely on the basis of HBsAg positivity could diagnose 28/77 cases that is 36.36% whereas using additional tests like anti-HBc IgG could further identify 36 cases. This increased the prevalence rate of HBV infection to 83.11% from 36.36% in CLD patients.

The prevalence of HCV infection in CLD patients has been reported to be between 10–40%[12,13] whereas in a high risk group like thalessemics the prevalence can be as high as 60%.[14] In the present study hepatitis C virus infection was seen in twelve cases that is in 15.58% as has been reported by others also. None of the HCV infection occurred in isolation and all the 12 cases were associated with evidence of past exposure to HBV. Our results support the observation that HCV infection occurs more often in association with HBV infection.[15]

All the 12 patients in our study who tested positive for anti-HCV were HBsAg negative. This is in accordance with other studies having reported the presence of HCV in serologically silent HBV infection with the prevalence ranging between 50–87%.[2–4] However in our study all the 12 cases i.e. 100% of the HCV infection were associated with serologically silent HBV infection.

The clinical significance of the silent HBV infection in HCV associated CLD has not been fully understood. A few studies have reported that the clinical course is generally more severe in patients with HBV / HCV co-infection,[6–8] whereas a few have reported decreased severity.[16] But in the present study presence of HCV co-infection did not alter the course of CLD as no significant change in the clinical presentation and biochemical parameters was observed in patients having isolated anti-HBc IgG positivity and those also having HCV co-infection. Isolated anti-HBc IgG positivity is highly prevalent in HCV infected individuals raising the possibility of potential interference between HBV and HCV replication. Since HBsAg could not be detected and HBV DNA was detected in only one case out of 12 anti-HCV positive cases it seems probable that HCV reduces HBV replication and thus exerts a stronger inhibitory effect on HBV. The above data clearly revealed the clearance/ suppression of HBV by HCV, which is in accordance with that reported by other workers.[17,18]

The patients having isolated HBc IgG and those having HCV co-infection did not have significantly different liver function profile but the deranged liver function test in patients having isolated HBc IgG positivity confirms the presence of liver disease in such patients.

## CONCLUSION

Thus it can be concluded from the present study that anti-HBc IgG could frequently be the only marker for past HBV infection and its presence should be tested in all patients of CLD as patients with isolated anti-HBc IgG positivity can develop liver disease. Furthermore patients showing isolated anti-HBc IgG should be monitored closely for liver function. These patients are also more likely to have presence of co-infection with HCV which should be actively searched for in them.
